# The Effect of Peripheral Immune Cell Counts on the Risk of Multiple Sclerosis: A Mendelian Randomization Study

**DOI:** 10.3389/fimmu.2022.867693

**Published:** 2022-05-10

**Authors:** Di He, Liyang Liu, Dongchao Shen, Peng Zou, Liying Cui

**Affiliations:** ^1^ Department of Neurology, Peking Union Medical College Hospital (PUMCH), Chinese Academy of Medical Sciences and Peking Union Medical College (CAMS & PUMC), Beijing, China; ^2^ Peking Union Medical College M.D. Program, Peking Union Medical College, Beijing, China; ^3^ Department of Cardiac Surgery, Beijing Tsinghua Changgung Hospital, Beijing, China; ^4^ Neuroscience Center, Chinese Academy of Medical Sciences and Peking Union Medical College (CAMS), Beijing, China

**Keywords:** multiple sclerosis, mendelian randomization (MR), genome wide association study (GWAS), peripheral immune cell count, NKT cell

## Abstract

**Objectives:**

Multiple sclerosis (MS) is a complex central nervous system (CNS) demyelinating disease, the etiology of which involves the interplay between genetic and environmental factors. We aimed to determine whether genetically predicted peripheral immune cell counts may have a causal effect on MS.

**Methods:**

We used genetic variants strongly associated with cell counts of circulating leukocyte, lymphocyte, monocyte, neutrophil, eosinophil, and basophil, in addition to some subpopulations of T and B lymphocyte, as instrumental variables (IVs) to perform Mendelian randomization (MR) analyses. The effect of immune cell counts on MS risk was measured using the summary statistics from the International Multiple Sclerosis Genetics Consortium (IMSGC) genome-wide association studies (GWAS).

**Results:**

Our findings indicated that higher leucocyte count [odds ratio (OR), 1.24; 95% confidence interval (CI), 1.07 - 1.43; *p* = 0.0039] and lymphocyte count (OR, 1.17; 95% CI, 1.01 – 1.35; *p* = 0.0317) were causally associated with MS susceptibility. In addition, we also found that increase of genetically predicted natural killer T (NKT) cell count is also associated with an increase MS risk (OR, 1.24; 95% CI, 1.06 - 1.45; *p* = 0.0082).

**Conclusions:**

These findings show that the genetic predisposition to higher peripheral immune cell counts can exert a causal effect on MS risk, which confirms the crucial role played by peripheral immunity in MS. Particularly, the causal association between NKT cell count and MS underscores the relevance of exploring the functional roles of NKT cells in disease pathogenesis in future.

## Introduction

As a chronic demyelinating disorder of central nervous system (CNS), multiple sclerosis (MS) is characterized by multiple disseminated inflammatory lesions both temporally and spatially (1). Although ultimately the patients will suffer from irreversible disability, the course of disease development is highly variable, featuring a wide range of focal neurological signs and symptoms that affect mobility, sensation or cognition (2). MS usually clusters within families but does not follow the Mendelian inheritance pattern (3). Such familial recurrence pattern indicates a polygenic risk model for MS, in which the risk is likely conferred by an allele of moderate effect size and several minor-effect alleles (4).

Notably, the genes implicated by these identified loci cluster in key immunological pathways involving lymphocyte activation, receptor signaling and cytokine production (5–7). In accordance, among the approved disease-modifying therapies for relapsing-remitting MS, many have immune modulatory effects, such as directing immune cell trafficking, suppressing auto-reactive T cells, inducing regulatory T cells (Tregs) and regulating B cell activities (8–12). Although their efficacy is expected to diminish with time, which possibly reflects the dwindling roles played by inflammation as the disease develops, early treatment with drugs that reshape the immune environment can indeed limit the rate of evolving to the secondary progressive stage (13). Therefore, the genetic architecture of MS susceptibility provides some solid evidence supporting the view that MS is an immune-mediated disease and highlights the prominent roles of immune dysregulation in MS predisposition.

Since genetic variants are fixed and randomly allocated at conception, Two-sample Mendelian randomization (MR) can exploit these variants as unbiased proxies to approximate the effect of an exposure on the outcome of interest while minimizing the effects of confounding and reverse causation (14, 15). Although both body mass index (BMI) and serum 25-hydroxyvitamin D [25(OH)D] have been consistently shown causally associated with MS *via* MR approach (16–18), few MR study has addressed the causal relationship between circulating immune cell counts and the disease. To further explore the causal roles played by peripheral immunity on MS risk, we implemented a two-sample MR analysis using recently published data from the largest GWAS to date on blood cell phenotypes (19) and a high-resolution immune cell profiling GWAS (20).

## Methods

### Exposure and Outcome Data Sources

Effect estimates for SNPs associated with peripheral blood cell counts, which include total leukocyte, lymphocyte, monocyte, neutrophil, eosinophil, and basophil, were obtained from the Blood Cell Consortium (BCX) meta-analysis, which includes data from 563,085 European ancestry individuals (19). For cellular subpopulation analyses, which include absolute cell counts of T cells [naïve, central memory (CM), effector memory (EM), terminally differentiated (TD), regulatory (Treg) and natural killer (NKT)] and B cells (naïve, unswitched memory, switched memory and transitional), we used GWAS summary statistics from Orru ` et al., which profiled 3,757 individuals by flow cytometry with the Sardinian founder population (20).

Effect estimates of these SNPs on the risk of MS were evaluated with GWAS summary statistics from the International Multiple Sclerosis Genetics Consortium (IMSGC) study, which involves 47,429 MS cases and 68,374 control subjects of European ancestry (21). To limit potential bias for MR analyses resulting from population stratification, the summary statistics was derived from individuals of European descent for both exposure and outcome datasets. Since the present study uses only publicly available GWAS summery statistics without attempting to identify individual-level data, ethical approval was not sought for.

### Instrumental Variable Selection

For the blood cell traits derived from the two GWAS datasets, genetic variants achieving genome-wide significance were selected at the p-value cutoff of 5 × 10^-8^. To guarantee that the variants used as instrumental variables (IVs) are independent, we clumped the SNPs (R^2^ < 0.001 with any other associated SNP within 10 kb window) based on 1000 Genomes Project linkage disequilibrium (LD) reference panel, with the SNP showing the lowest *p* value at each locus retained. Given the modest scale of the second GWAS (20), a more relaxed clumping threshold (R^2^ < 0.01) was used. The association statistics of these genetic variants with MS were then extracted. Because the IV exclusion restriction assumption is unprovable in practice (22), we removed the variants showing potential pleiotropic association with MS (association *p* value lower than the genome-wide suggestive significance level of 10^-5^), a conservative strategy allowing us to perform MR analysis with more confidence (23). To prevent the effect estimates from aligning with different allele, harmonization was performed to remove ambiguous SNPs showing non-concordant alleles. The maximum minor allele frequency (MAF) threshold for aligning palindromic SNPs was set for 0.3. When an exposure-associated SNP was not present in the outcome dataset, a proxy SNP highly correlated with the variant of interest (r^2^ > 0.8) was selected instead. Since previous MR studies have only confirmed that 25(OH)D and BMI are causally associated with MS (16, 17, 24), and there is a lack of evidence regarding other MS risk factors such as smoking and exposure to Epstein-Barr virus (EBV) (18), to meet the independence assumption of MR analysis, SNPs showing suggestive association (P < 10^-5^) with 25(OH)D or BMI were removed.

To quantitatively measure the strength of the selected SNPs, proportion of variance explained (PVE) by each IV was calculated with PVE = 2 × EAF × (1 − EAF) × β^2^ (EAF, effect allele frequency; β, effect size on the exposure), and F statistic for each IV was then calculated *via* [PVE × (n – 1 – k)]/[(1 – PVE) × k], where n represents the effective sample size in the exposure GWAS, and k represents the number of variants included in the IV model (25). Power estimation was performed using a web-tool (https://shiny.cnsgenomics.com/mRnd/) given a Type-I error rate α of 0.05 and the estimated OR from the IVW method.

**Figure 1:**
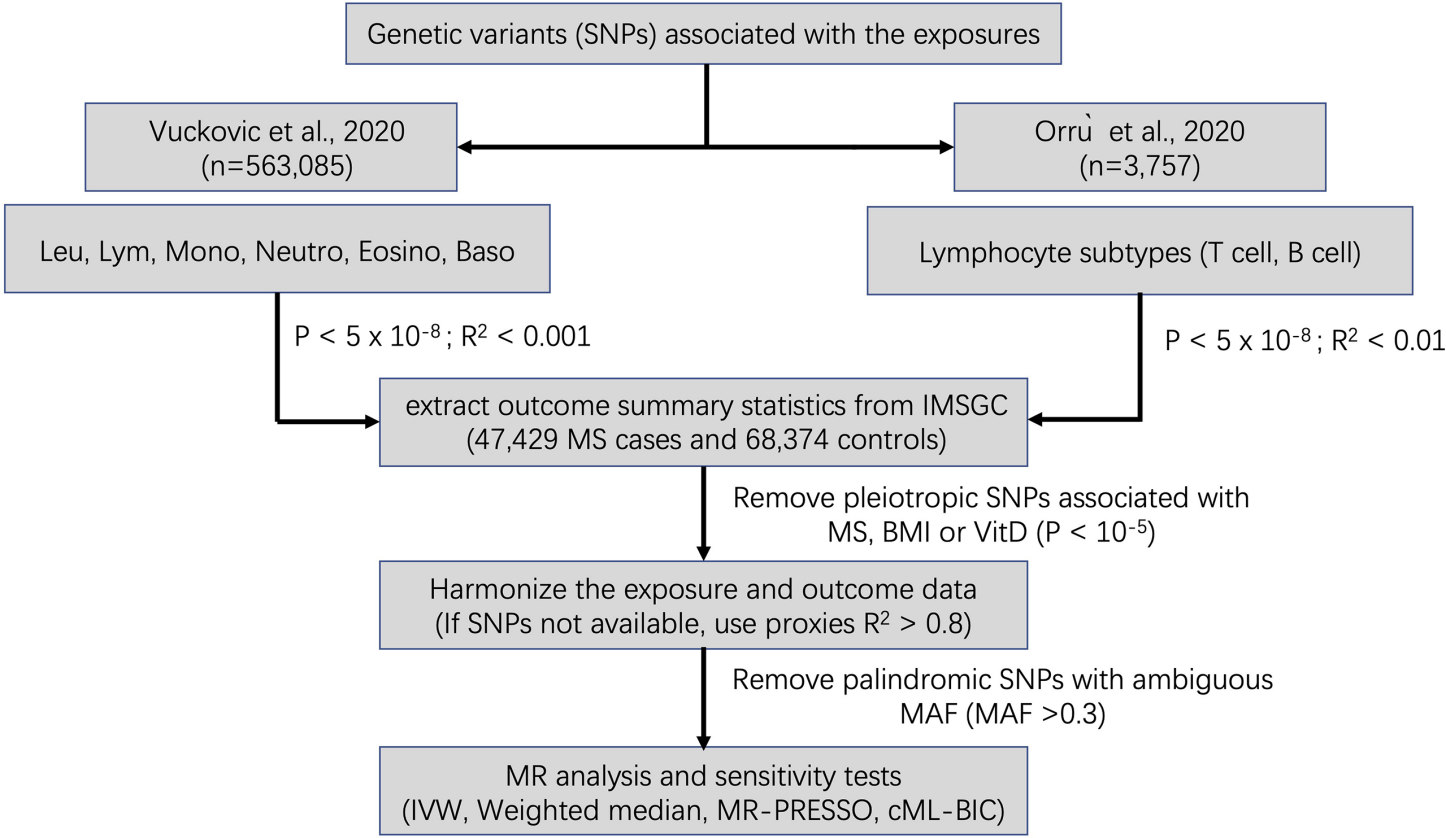
Schematic diagram for the Mendelian randomization analysis exploring effects of blood cell counts on multiple sclerosis. SNP, single nucleotide polymorphism; Leu, leukocyte; Lym, lymphocyte; Mono, monocyte; Neutro, neutrophil; Eosino, Eosinophil; Baso, basophil; IMSGC, International Multiple Sclerosis Genetics Consortium; IVW, Inverse variance weighted; MR-PRESSO, Mendelian Randomization Pleiotropy RESidual Sum and Outlier; cML-BIC, constrained maximum likelihood- Bayesian information criterion.

A flowchart summarizing our selection of IVs is displayed in **Figure 1**. The summary information for IVs used for MR analyses after clumping and data harmonization can be found in **Supplementary Tables 1–10**.

### Statistical Analyses

The inverse variance weighted (IVW) method, which meta-analysed individual Wald-type ratios of IV under a multiplicative random effects model, was selected as one of the principal two-sample MR analyses to estimate causal effects, with odd ratios (ORs) described as per standard deviation (SD) increase in the levels of risk factor (26). Heterogeneity of IVs was assessed *via* the Cochran’s Q test. Because the IVW estimate is based on the no measurement error (NOME) assumption, both weighted median approach and MR pleiotropy residual sum and outlier (MR-PRESSO), which adopt a more lenient majority-valid or InSIDE (instrument strength independent of direct effect) assumption, were incorporated into the analyses to account for the presence of potential pleiotropy (27, 28). In addition, a recently described sensitivity test, constrained maximum likelihood and model averaging and Bayesian information criterion (cML-MA-BIC), was used to address potential violation of IV assumptions (29). The causal effect of an exposure on MS is considered indicative if the effect estimate is nominally significant in the IVW method and no contradictory results are found in the sensitivity analyses. Bonferroni correction was used to adjust for multiple testing. Potential directional pleiotropy was evaluated by MR-Egger regression intercept (30). Leave-one-out (LOO), which implements a sampling strategy, were used to detect IV outliers substantially influencing causal effects (31). Finally, funnel plots and scatter plots were evaluated as visual inspection of symmetry and the effect estimates. To assess whether the exposure to MS may have causal impacts on the absolute counts of immune cells, bi-directional MR was conducted using the primary non-MHC SNPs associated with MS (21).

The analyses were carried out using the TwoSampleMR (version 0.5.6), MR-cML (version 0.0.0.9) and MR-PRESSO (version 1.0) packages implemented in R (version 3.4). The forest plots were drawn using Forestplot package (Version 2.0.1).

## Results

**Figure 2:**
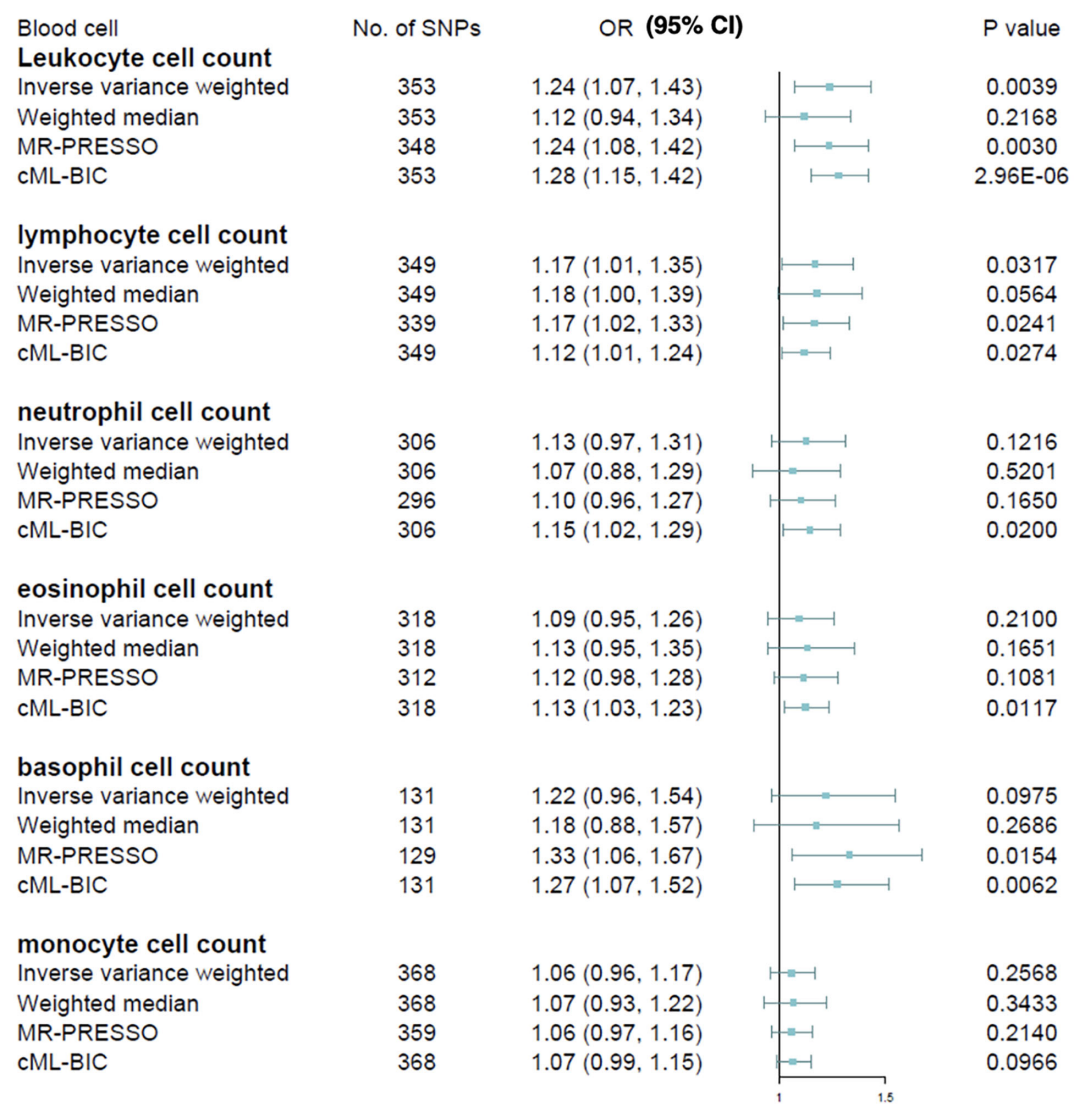
Mendelian randomization estimates of the association between blood cell counts and risk of multiple sclerosis. OR, odds ratio; CI, confidence interval.

The causal estimates of immune cell count on MS risk are as summarized graphically in **Figure 2**. We observed that higher leucocyte count was robustly associated with increased MS susceptibility using IVW method [odds ratio (OR), 1.24; 95% confidence interval (CI), 1.07 – 1.43; *p* = 0.0039] after correcting for multiple testing (*p* < 0.05/6 for the six immune cell traits). Among the five major leucocyte subtypes, we also found suggestive evidence that lymphocyte count was positively associated with disease risk (OR, 1.17; 95% CI, 1.01 – 1.35; *p* = 0.0317). Sensitivity analyses provided consistent results and did not suggest bias from genetic pleiotropy. LOO analysis did not give evidence of SNPs disproportionally affecting the effect estimates. No obvious directional pleiotropy can be detected by visual inspection of funnel plots, and the MR-Egger regression intercept was also insignificant. The results from heterogeneity test, pleiotropy test and F-statistic is summarized in **Supplementary Table 11**. Because significant heterogeneity was detected by Cochran Q test in some cell types, the random effect model was used to estimate the MR effect size (32). In contrast, no significant association was observed for neutrophil, basophil, eosinophil, or monocyte cell counts with MS, although a trend of positive correlation can be observed.

**Figure 3:**
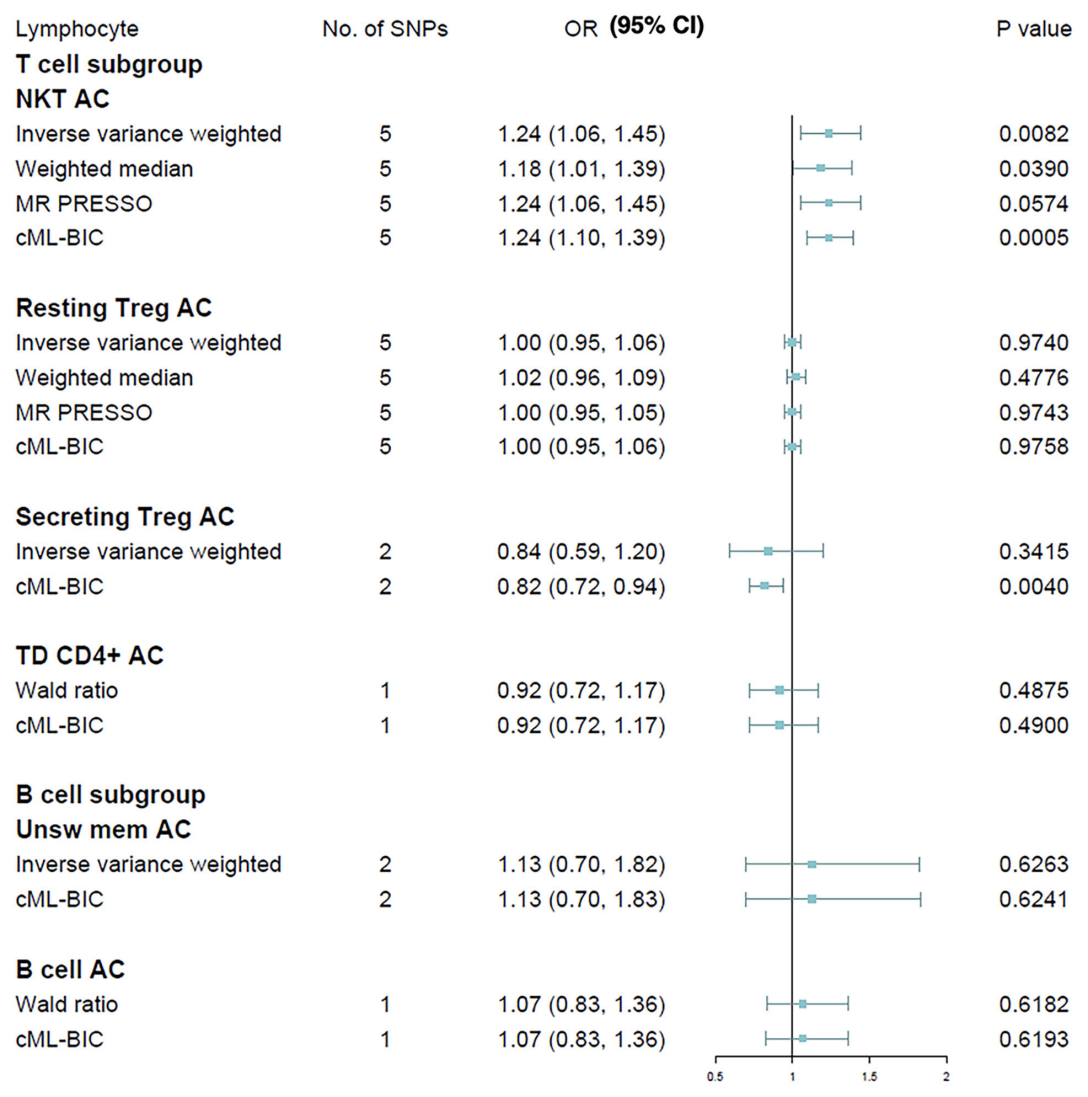
Mendelian randomization results for the relationship between cell counts of lymphocyte subpopulation and multiple sclerosis. AC, absolute count.

We next extended our analyses by further measuring the causal estimates of T cell and B cell on MS risk, the two major lymphocyte subpopulations implicated with the disease. T lymphocytes were gated based on the expression of CD3^+^ (total), CD45RA^+^CCR7^+^ (naive), CCR7^+^CD45RA^−^ (CM), CD45RA^−^CCR7^−^ (EM), CCR7^−^CD45RA^+^ (TD) and CD16/CD56^+^ (NKT). Tregs (CD25^hi^CD127^lo^) were subdivided into activated (CD25^+++^CD45RA^−^), resting (CD25^++^CD45RA^+^) and secreting (CD25^++^CD45RA^−^). B lymphocytes were gated based on the expression of CD19^+^ (total), CD24^+^CD38^hi^ (transitional), CD24^−^CD38^−/dim^ (naive), CD27^+^IgD^−^ (switched memory) and CD27^+^IgD^+^ (unswitched memory). Due to the small GWAS sample size, currently we were only able to assess six of these lymphocyte subpopulations *via* MR methods, i.e., NKT, resting Treg, secreting Treg, TD T cell, B cell and unswitched memory B cell. Results of Wald ratio are presented instead of IVW in cases where less than two IVs is available, and only IVW and cML-BIC are shown when there are less than three IVs (**Figure 3**). The IVW method revealed that an increase of NKT cell count was associated with a higher risk of MS (OR, 1.24; 95% CI, 1.06 - 1.45; *p* = 0.0082), which was supported by other MR methods. The causal effects of the other cellular subtypes on MS risk were attenuated to null. We further analyzed the two NKT subsets (CD8^br^ and CD8^dim^) for which qualifying IV(s) is available. The results indicated that neither CD8^br^ NKT (OR, 1.05; 95% CI, 0.83 - 1.33; *p* = 0.70) nor CD8^dim^ NKT (OR, 1.05; 95% CI, 0.95 - 1.17; *p* = 0.34) subset was causally associated with MS. In addition, genetic predisposition to MS as exposure did not have causal impact on the absolute counts of leukocyte (OR, 1.01; 95% CI, 0.99 - 1.03; *p* = 0.38), lymphocyte (OR, 1.01; 95% CI, 0.98 - 1.04; *p* = 0.39) or NKT cell (OR, 1.00; 95% CI, 0.95 - 1.06; *p* = 0.88) in bi-directional MR analyses.

## Discussion

Epidemiologic and genetic studies have implied correlation between peripheral immunity and MS (3, 21, 33). Since most of the GWAS variants identified are noncoding variants located in the regulatory region of genes, elucidating their functional consequences can be extremely challenging (34, 35). Moreover, due to reverse causation and confounding, drawing causal inferences is also difficult and liable to bias. In comparison, the MR approach, which uses genetic variants robustly associated with the exposure as IVs, can be used to infer causality while controlling for various sources of confounder. In the present study, we first found evidence that peripheral leucocyte and lymphocyte counts were positively associated with the risk for MS using summary statistics from a recent large scale GWAS analyzing blood cell traits. This is in line with some recent MR studies, which indicate a causal relation between circulating interleukins (ILs) and MS (36), and that the association between BMI and MS is partly mediated *via* IL-6 signaling (37). Considering that the regulatory effects of variants are often dependent on cellular subtypes, it is to be expected that deciphering their pathologic roles will require analyzing the disease-relevant cell types (6, 38). We thus hypothesize that MS might be caused by the dysfunction of a relatively minor proportion of total lymphocytes, in which more prominent effects can be found. We further analyzed several subtypes of B cells and T cells, the two major cellular subtypes within lymphocyte population known to influence MS progression. We found that NKT cells were causally associated with an increased risk for MS, although no significant association was found by subdividing NKT cells based on CD8 expression.

NKT cells, which are innate-like T cells sharing features with both adaptive and innate cells, can be activated rapidly in response to self or foreign lipid antigens and adopt proinflammatory or immunoregulatory phenotypes in mice and human (39). In keeping with their modulatory functions, it has been noted that their levels are lower in the peripheral blood of MS patients (40), which can be restored during remission in patients undertaking IFN-β treatment (41). However, whether the lower percentage of circulating NKT cells indicates that they are protective in MS or whether it is only reflective of disease progression remains controversial. Such complexity can be partly explained by the heterogeneity of this cell population. NKT cells can be broadly divided into two distinct populations based on their expression of T cell receptor (TCR): the classical type I NKT (or invariant NKT, iNKT) and the non-classical type II NKT (42). Like CD4^+^ T cells, which can be categorized into Th1, Th2 or Th17 based on their cytokine secretion profile when activated, polarization towards distinctive functional subsets has also been described for iNKT cells (43).

iNKT cells can be induced by lipid and glycolipid antigens presented on the MHC Class I-related molecule CD1d, such as alpha-galactosylceramide (α-GalCer), to modulate experimental autoimmune encephalomyelitis (EAE) by secreting mixtures of cytokines typically associated with either pro-inflammatory [such as interferon gamma (IFN-γ)] or anti-inflammatory [such as interleukin 4 (IL-4)] responses (44). Of note, a synthetic glycolipid (OCH), which can induce a predominant production of IL-4 by iNKT cells, has been described, as it is particularly efficient for suppressing EAE *via* eliciting a Th2-biased cytokine production profile (45). Similarly, using transcriptomic and functional analysis, Carrion et al. have reported that the iNKT cells reactive to a human collagen type II self-peptide (hCII707-721) may constitute a protective iNKT cell subset, which is absent in primary and secondary progressive MS patients (46). On the other hand, iNKT cells also play a prominent role in B cell maturation and activation *via* CD40-CD40L, CD1d-TCR interaction and IFN-γ/IL-4 production (47). Given the efficiency of selective B cell depletion therapies in treating MS with anti-CD20 monoclonal antibodies (10, 11) and the increasingly recognized roles played by B cells in MS immunopathology (48), it is inconclusive whether iNKT cells may have predominantly protective or detrimental effects in MS.

Unlike in mice, circulating iNKT cells normally constitute less than 0.1% of total lymphocytes in human, which are outnumbered by type II NKT cells (49). It has been shown that type II NKT cells can react with sulfatide, a sulfated glycolipid enriched in the myelin sheaths that insulate nerve fibers, and that type II NKT cells, but not iNKT cells, accumulated in the CNS in the EAE model (50). Consistently, these sulfatide-reactive T cells were also more common in the peripheral blood of MS patients than controls (51), which further support the possibility that type II NKT cells contribute to MS pathogenesis. However, since type II NKT cells are harder to identify in human samples due to a lack of specific surface markers like α- GalCer/CD1d tetramers, they are much less studied than iNKT cells, and their roles in autoimmunity remain to be further validated (52).

Our study is subject to several limitations. First, as noted above, the NKT cells are a heterogenous population, whereas we were unable to distinguish between Type I and Type II NKT cells for our MR analyses, as the cells were gated as CD3^+^CD16^+^/CD56^+^ in their original flow panel, and only CD8 expression level was used to subdivide NKT population (20). In addition, NKT cells are not the only populations that co-express these molecules in blood, with Yδ T cells also sharing this co-expression (53). Therefore, it is necessary to refine cell populations with panels focusing on NKT cells to solidify and expand our findings. Second, given the relatively modest scale of the lymphocyte phenotyping GWAS, our second part of the MR analyses were limited by the availability of the variants that can be effectively used as IVs. Granted, relaxing the association threshold to a *p* value of < 10^-5^, as was adopted by Orru ` et al. in their MR analyses (20), would undoubtedly provide more IVs, this would also compromise the first MR assumption (i.e., the variant being robustly associated with the exposure of interest). With the advent of larger scale phenotyping GWAS in future, we expect more SNPs passing GWAS significance threshold that can be reliably used as IVs for MR analysis. Third, although Mediterranean Sardinian population has been widely used for genetic studies to identify causal variants in complex diseases such as MS (54), it is relatively isolated from the mainland European population. Sardinia is colonized by the Neolithic Early European Farmers (EEF) with minor contributions from pre-Neolithic West European hunter-gatherers (WHG), two of the three ancestral populations that contribute ancestry to the present-day European populations (the other being the Ancient North Eurasians, ANE), and thus Sardinian population shares ancestral connection with part of Spanish and French populations (55, 56). Given the indicative causal association between NKT cell count and MS risk presented here, further genetic profiling using diverse European population is recommended. Finally, unlike the first MR assumption, the other two assumptions are not fully testable in practice. Because using variants that influence other traits outside the pathway of interest or that have a direct effect on the target outcome as IVs can potentially distort MR analysis and give false-positive causal inference, we applied several sensitivity tests to evaluate the robustness of the results, including MR-PRESSO and cML-MA-BIC to detect and adjust for horizontal pleiotropic outliers and violation of MR assumptions. By comparing results from different MR methods, we were able to minimize the potential bias resulting from pleiotropic effects, although careful interpretation of the causal inference is still warranted.

## Conclusions

This study provides evidence that higher circulating leucocyte and lymphocyte counts increase the risk of MS. In addition, we also found causal association between higher NKT cell count and MS, which underscores the relevance of exploring the functional roles of NKT cells in MS. Given that type II NKT cells are more abundant and have a diverse immunoregulatory roles in human, we have great expectation that progress in understanding this T cell subset may hold promising immunotherapeutic potential.

## Data Availability Statement

The original contributions presented in the study are included in the article/**Supplementary Material**. Further inquiries can be directed to the corresponding authors.

## Ethics Statement

Ethical review and approval was not required for the study on human participants in accordance with the local legislation and institutional requirements. Written informed consent for participation was not required for this study in accordance with the national legislation and the institutional requirements.

## Author Contributions

DH: study design and conceptualization, data interpretation, manuscript drafting for intellectual content. LL: data analysis and interpretation, manuscript revision. DS: data interpretation, manuscript revision. PZ: data interpretation, manuscript revision. LC: study design and conceptualization, data interpretation, manuscript revision. All authors contributed to the article and approved the submitted version.

## Funding

This work was supported by the Strategic Priority Research Program (Pilot study) “Biological basis of aging and therapeutic strategies” of the Chinese Academy of Sciences (Grant number: XDB39040000).

## Conflict of Interest

The authors declare that the research was conducted in the absence of any commercial or financial relationships that could be construed as a potential conflict of interest.

## Publisher’s Note

All claims expressed in this article are solely those of the authors and do not necessarily represent those of their affiliated organizations, or those of the publisher, the editors and the reviewers. Any product that may be evaluated in this article, or claim that may be made by its manufacturer, is not guaranteed or endorsed by the publisher.
